# Supplementary material for: The Influence of Graphene Oxide-Fe3O4 Differently Conjugated with 10-Hydroxycampthotecin and a Rotating Magnetic Field on Adenocarcinoma Cells
Source: Int J Mol Sci. 2024 Jan 11;25(2):930. doi: 10.3390/ijms25020930 (PMC10816047; doi:10.3390/ijms25020930)
Supplement: Supplementary file 1 [file ijms-25-00930-s001.zip › ijms-2776466-supplementary.pdf]

*Supporting Information*

**The influence of graphene oxide – $Fe_3O_4$ differently conjugated with 10-hydroxycampthotecin and rotating magnetic field on adenocarcinoma cells**

Magdalena Jedrzejczak-Silicka[1]*†, Karolina Szymańska[2]†, Ewa Mijowska[2], Rafał Rakoczy[3]*

[1]Laboratory of Cytogenetics, West Pomeranian University of Technology, Szczecin, Klemensa Janickiego 29, 71-270 Szczecin Poland

[2]Department of Physicochemistry of Nanomaterials, Faculty of Chemical Technology and Engineering, West Pomeranian University of Technology, Szczecin, Piastow Ave. 42, 71-065 Szczecin, Poland

[3]Institute of Chemical Engineering and Environmental Protection Process, West Pomeranian University of Technology, Szczecin, Piastow Avenue 42, 71-065 Szczecin, Poland

*Corresponding authors:

1. Magdalena Jedrzejczak-Silicka, Email: mjedrzejczak@zut.edu.pl

Laboratory of Cytogenetics, Faculty of Biotechnology and Animal Husbandry, West Pomeranian University of Technology, Klemensa Janickiego 29, 71-270 Szczecin, Poland

2. Rafał Rakoczy, Email: rrakoczy@zut.edu.pl

Institute of Chemical Engineering and Environmental Protection Process, West Pomeranian University of Technology, Szczecin, Piastow Avenue 42, 71-065 Szczecin, Poland

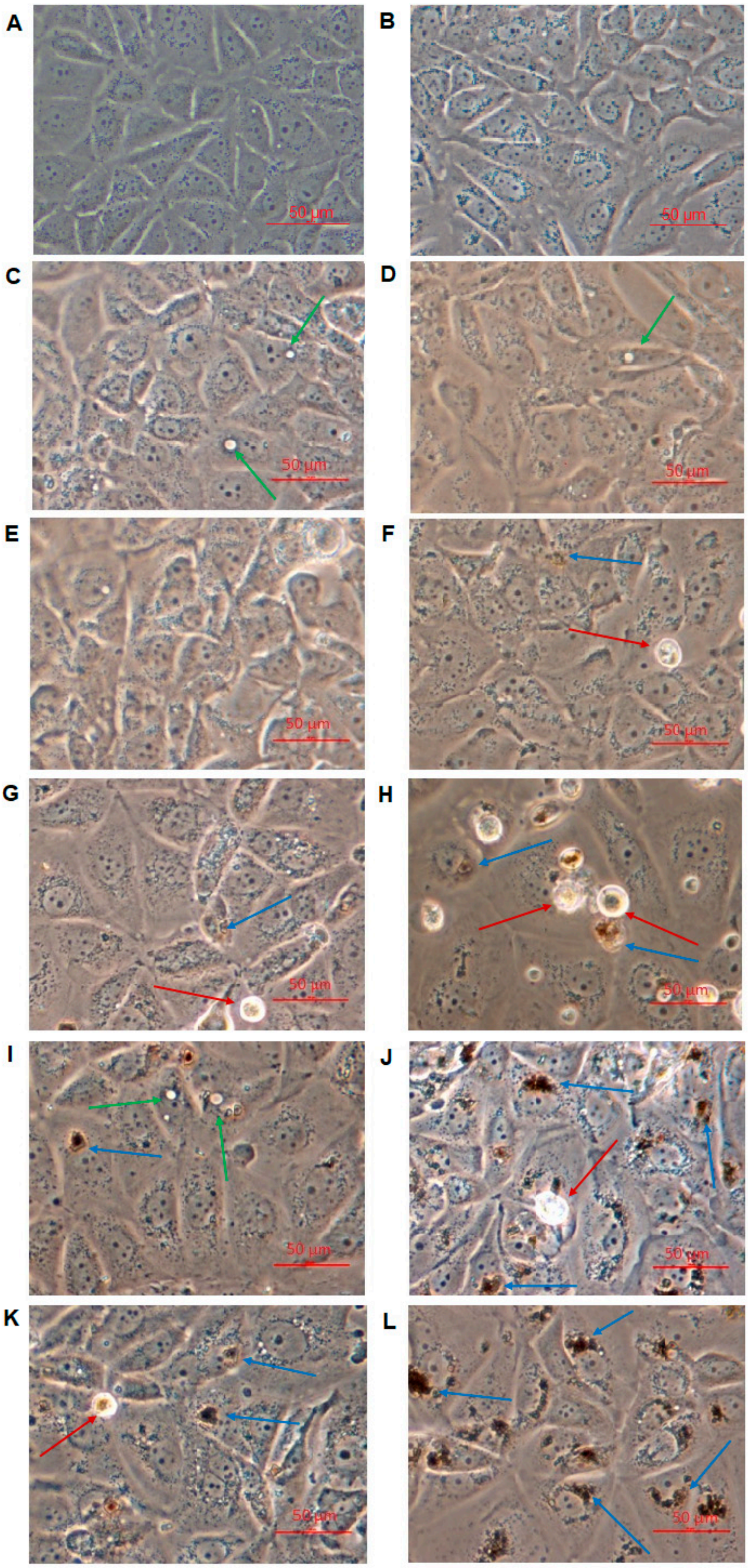


**Figure S1.** MCF-7 cell line morphology after 8 hours incubation in rotating magnetic field in (a-b) control culture; (c) cultures incubated with c-GO-$Fe_3O_4$-HCPT and (d) with nc-GO-$Fe_3O_4$-HCPT at concentration 3.125 $\mu g \cdot ml^{-1}$; (e) MCF-7 cells exposed to c-GO-$Fe_3O_4$-HCPT and (f) to nc-GO-$Fe_3O_4$-

HCPT at concentration 6.25 μg·ml$^{-1}$; (g) MCF-7 cultures incubated with c-GO-$Fe_3O_4$-HCPT and (h) with nc-GO-$Fe_3O_4$-HCPT at concentration 12.5 μg·ml$^{-1}$; (i) cells exposed to c-GO-$Fe_3O_4$-HCPT and (j) to nc-GO-$Fe_3O_4$-HCPT at concentration 25.0 μg·ml$^{-1}$; (k) cultures incubated with c-GO-$Fe_3O_4$-HCPT and (l) with nc-GO-$Fe_3O_4$-HCPT at concentration 50.0 μg·ml$^{-1}$.

Green arrows indicate vacuoles; blue arrows show shrunken cells; red arrows indicate nanomaterials accumulated within cells or aggregates.

**Table S1.** Statistically significant differences between means obtained from MCF-7 cell cultures incubated with graphene oxide-$Fe_3O_4$ analyzed using WST-1, LDH and NR assays (small letters present differences considered as significant at a level of $p<0.05$).

| | Concentration (µg/mL) | | | | | | | | | | | | | | | | | |
|---|---|---|---|---|---|---|---|---|---|---|---|---|---|---|---|---|---|---|
| | 0 | | | 3.125 | | | 6.25 | | | 12.5 | | | 25.0 | | | 50.0 | | |
| | a | | | b | | | c | | | d | | | e | | | f | | |
| | WST-1 | LDH | NR | WST-1 | LDH | NR | WST-1 | LDH | NR | WST-1 | LDH | NR | WST-1 | LDH | NR | WST-1 | LDH | NR |
| GO-$Fe_3O_4$ | c | d | b, c, d, e, f | c, e, f | d | a | a, b, d, e, f | - | a | c, e, f | a, b | a | b, c, d | - | a | b, c, d | - | a |

**Table S2.** Statistically significant differences between means obtained from MCF-7 cell cultures incubated with different concentration of hydroxycamptothecin analyzed using WST-1, LDH and NR assays (small letters present differences considered as significant at a level of $p<0.05$).

| | Concentration (µg/mL) | | | | | | | | | | | | | | | | | | | | |
|---|---|---|---|---|---|---|---|---|---|---|---|---|---|---|---|---|---|---|---|---|---|
| | 0 | | | DMSO | | | 3.125 | | | 6.25 | | | 12.5 | | | 25.0 | | | 50.0 | | |
| | a | | | b | | | c | | | d | | | e | | | f | | | g | | |
| | WST-1 | LDH | NR | WST-1 | LDH | NR | WST-1 | LDH | NR | WST-1 | LDH | NR | WST-1 | LDH | NR | WST-1 | LDH | NR | WST-1 | LDH | NR |
| HCPT | b, c, d, e, f, g | b, c, d, e, f, g | b, c, d, e, f, g | a, c, d, e, f, g | a, c, d, e, f, g | a, c, d, e, f, g | a, b | a, b | a, b, d | a, b | a, b | a, b, c, f, g | a, b | a, b | a, b, c | a, b | a, b | a, b, d | a, b | a, b | a, b, d |

**Table S3.** Statistically significant differences between means of MCF-7 cell controls and experimental samples exposed to different magnetic induction intensity of RMF obtained from WST-1, LDH and NR assays (small letters present differences considered as significant at a level of $p<0.05$).

| | Magnetic induction (mT) | | | | | | | | | | | | | | | | | | | | |
|---|---|---|---|---|---|---|---|---|---|---|---|---|---|---|---|---|---|---|---|---|---|
| | 0 (without RMF) | | | 1.23 | | | 1.57 | | | 2.36 | | | 3.95 | | | 6.58 | | | 10.06 | | |
| | a | | | b | | | c | | | d | | | e | | | f | | | g | | |
| | WST-1 | LDH | NR | WST-1 | LDH | NR | WST-1 | LDH | NR | WST-1 | LDH | NR | WST-1 | LDH | NR | WST-1 | LDH | NR | WST-1 | LDH | NR |
| 8 h exposition | e, f, g | b, c, d, g | a | f, g | a, e, f, g | a | g | a, e, f, g | a | g | a, f, g | a | a | b, c, g | a | a, b | b, c, d, g | a | a, b, c, d | a, b, c, d, e, f | a, b, c, d, e, f |
| 24 h after exposition | e, f, g | g | a | - | g | a | - | g | a | - | g | a | a | g | a | a | g | a | a | a, b, c, d, e, f | a, b, c, d, e, f |

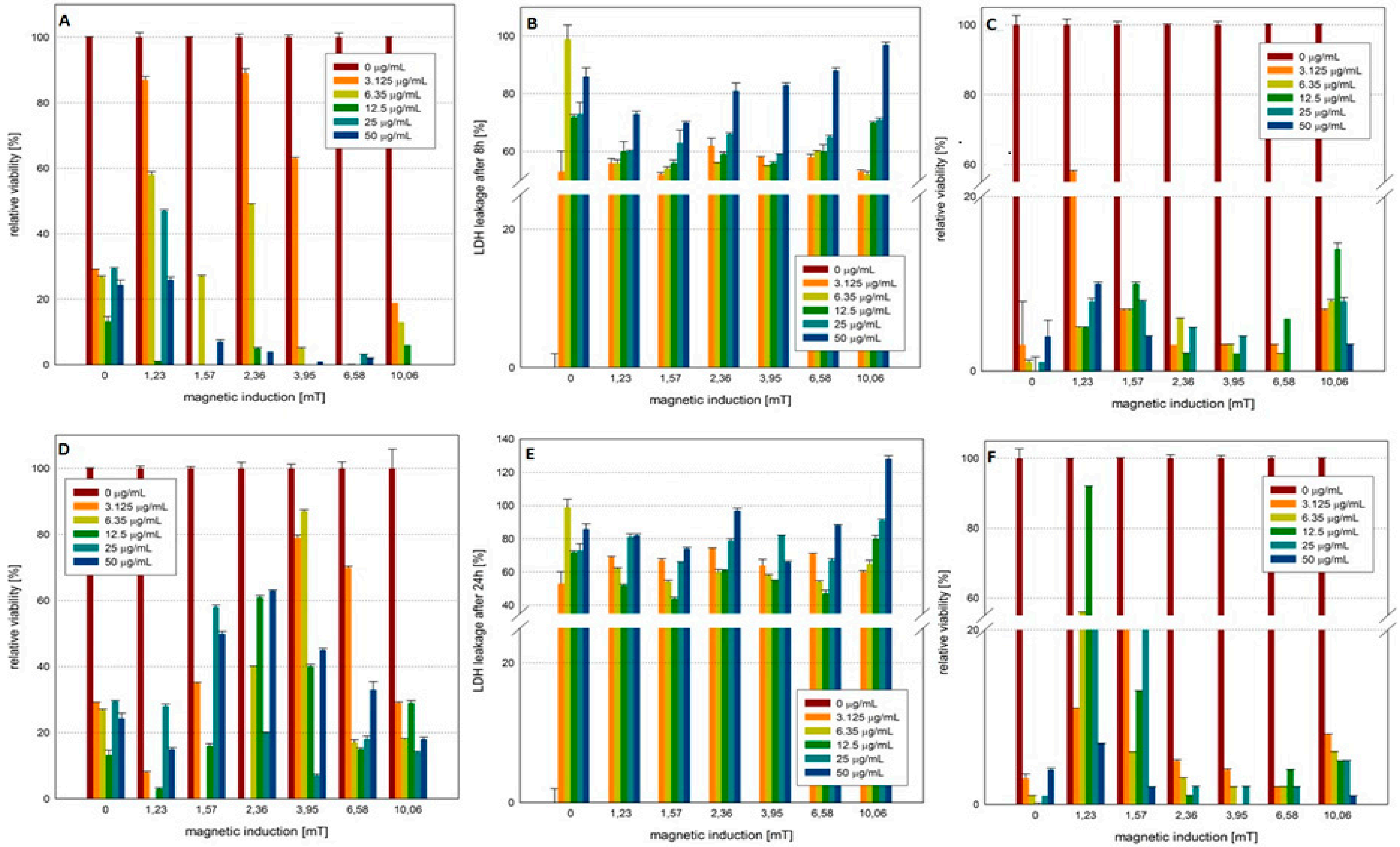


**Figure S2.** The response of MCF-7 cells to c-GO-$Fe_3O_4$-HCPT nanomaterial and RMF; (a) relative viability (WST-1 assay results); (b) LDH membrane leakage (LDH assay results); (c) neutral red uptake (NR assay results); (d) relative viability 24-hour after RMF exposure (WST-1 assay); (e) LDH release 24-hour after RMF exposure (LDH assay); (f) NR uptake 24-hour after RMF exposure (p-values<0.05 are considered significant and are represented by small letters, Table S4).

**Table S4.** Statistically significant differences between means of MCF-7 cell controls and experimental samples exposed to different magnetic induction intensity of RMF obtained from WST-1, LDH and NR assays (small letters present differences considered as significant at a level of $p<0.05$).

| Type of nanomaterial | Magnetic induction (mT) | | | | | | | | | | | | | | | | | | | | |
|---|---|---|---|---|---|---|---|---|---|---|---|---|---|---|---|---|---|---|---|---|---|
| | 0 (without RMF) | | | 1.23 | | | 1.57 | | | 2.36 | | | 3.95 | | | 6.58 | | | 10.06 | | |
| | a | | | b | | | c | | | d | | | e | | | f | | | g | | |
| | WST-1 | LDH | NR | WST-1 | LDH | NR | WST-1 | LDH | NR | WST-1 | LDH | NR | WST-1 | LDH | NR | WST-1 | LDH | NR | WST-1 | LDH | NR |
| c-GO-$Fe_3O_4$-CPT 8 h | c, d, f, g | b, c, d, e, f, g | b, c, d, e, f, g | c, d, e, f, g | a | a | a, b, d, e, f, g | a | a | a, b, c, g | a | a | b, c, g | a | a | a, b, c, g | a | a | a, b, c, d, e, f | a | a |
| c-GO-$Fe_3O_4$-CPT 24 h | c, f, g | b, c, d, e, f, g | b, c, d, e, f | c, f, g | a, g | a, d, e, f, g | a, b, d, e, f, g | a, g | a, d, e, f, g | c, f, g | a, g | a, b, c, e, f, g | c, f, g | a, g | a, b, c, e, f, g | a, b, c, d, e, g | a, g | a, b, c, d, e | a, b, c, d, e, f | a, b, c, d, e, f | b, c, d, e |
| nc-GO-$Fe_3O_4$-CPT 8 h | b, c, d, e, f, g | b, c, d, e, f, g | b, c, d, e, f, g | a, c, d, e, f, g | a | a | a, b, f, g | a | a | a, b, f, g | a | a | a, b, f, g | a | a | a, b, c, d, e, g | a | a | a, b, c, d, e, f | a | a |
| nc-GO-$Fe_3O_4$-CPT 24 h | b, c, d, e, f, g | b, c, d, e, f, g | d, e, f, g | a, g | a | d, e, f, g | a, g | a, d, e, f | d, e, f, g | a, g | a, c | a, b, c, e | a, g | a, c | a, b, c, d, f | a, g | a, b, c, e | a, c | a, b, c, d, e, f, | a | a, b, c |

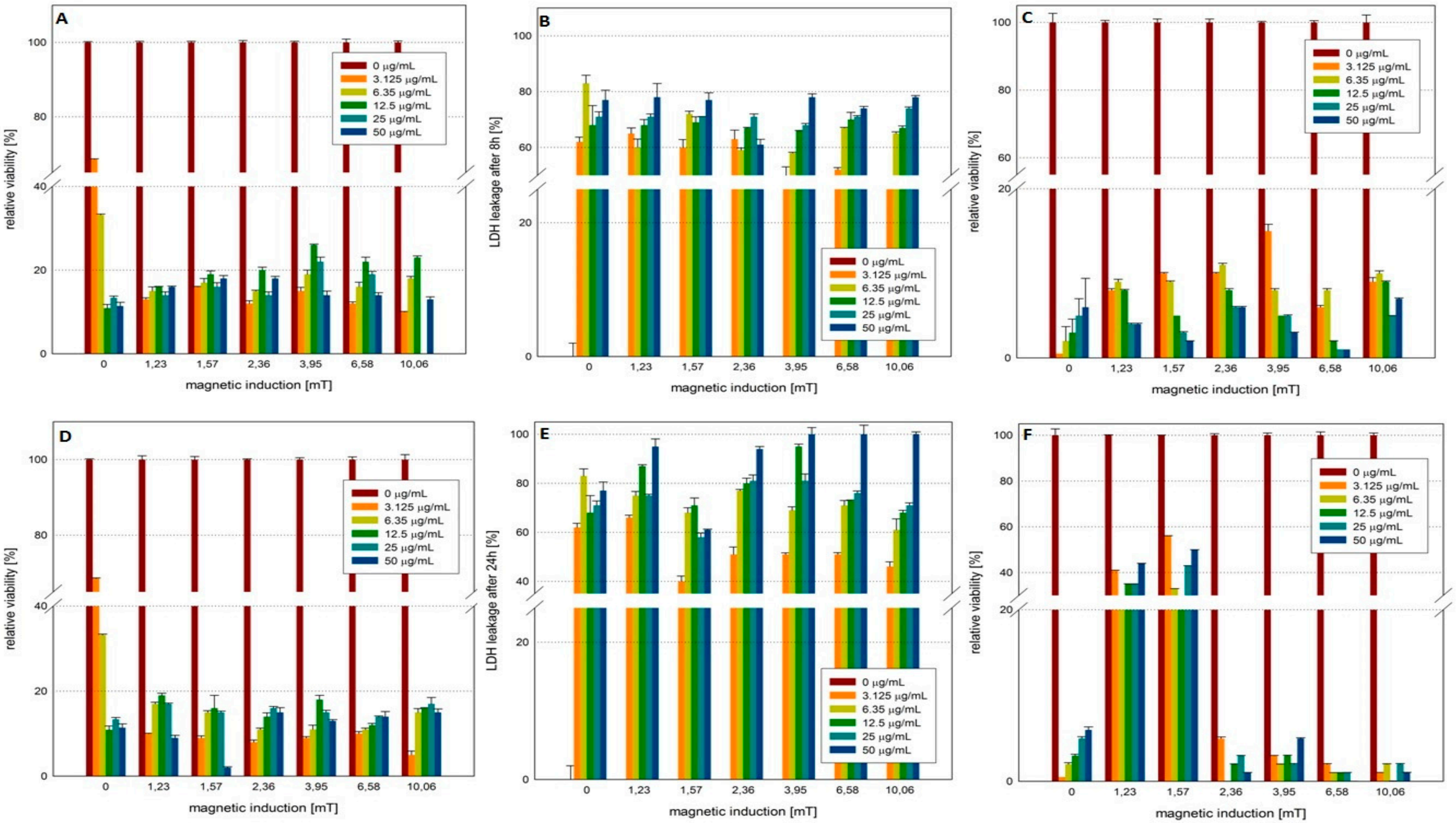


**Figure S3.** The response of MCF-7 to nc-GO-$Fe_3O_4$-HCPT nanomaterial and RMF; (a) relative viability (WST-1 assay results); (b) LDH membrane leakage (LDH assay results); (c) neutral red uptake (NR assay results); (d) relative viability 24-hour after RMF exposure (WST-1 assay results); (e) LDH release 24-hour after RMF exposure (LDH assay results); (f) NR uptake assay 24-hour after RMF exposure (p-values<0.05 are considered significant and are represented by small letters, Table S5).

**Table S5.** Statistically significant differences between means of MCF-7 cell controls and experimental samples exposed to different magnetic induction intensity of RMF obtained from WST-1, LDH and NR assays (small letters present differences considered as significant at a level of $p<0.05$).

| Type of nanomaterial | NPs concentration (µg/mL) | | | | | | | | | | | | | | | | | |
|---|---|---|---|---|---|---|---|---|---|---|---|---|---|---|---|---|---|---|
| | 0 (without NPs) | | | 3.125 | | | 6.25 | | | 12.5 | | | 25.0 | | | 50.0 | | |
| | a | | | b | | | c | | | d | | | e | | | f | | |
| | WST-1 | LDH | NR | WST-1 | LDH | NR | WST-1 | LDH | NR | WST-1 | LDH | NR | WST-1 | LDH | NR | WST-1 | LDH | NR |
| c-GO-$Fe_3O_4$-CPT 8 h | b, c, d, e, f | b, c, d, e, f | b, c, d, e, f | a, c, d, e, f | a, f | a | a, b, d, e | a, f | a | a, b, c, f | a, f | a | a, b, c, f | a | a, f | a, b, d, e | a, b, c, d, e | a |
| c-GO-$Fe_3O_4$-CPT 24 h | b, c, d, e, f | b, c, d, e, f | b, c, d, e, f | a, c, d, e, f | a, d, f | a, f | a, b, e | a, b, f | a | a, b, f | a, b, e, f | a | a, b, c, f | a, c, d, f | a | a, b, d, e | a, b, c, d, e | a, b |
| nc-GO-$Fe_3O_4$-CPT 8 h | b, c, d, e, f | b, c, d, e, f | b, c, d, e, f | a, c, d | a, d, e, f | a | a, b, e, f | a | a | a, b, e, f | a, b | a | a, c, d | a, b | a | a, c, d | a, b | a |
| nc-GO-$Fe_3O_4$-CPT 24 h | b, c, d, e, f | b, c, d, e, f | b, c, d, e, f | a, c, d, e | a, c, d, e, f | a | a, b, f | a, b, f | a | a, b, f | a, b, f | a | a, b, f | a, b, f | a | a, c, d, e | a, b, c, d, e | a |
